# Failed Weaning from Mechanical Ventilation and Cardiac Dysfunction

**DOI:** 10.1155/2012/173527

**Published:** 2012-09-06

**Authors:** Jahan Porhomayon, Peter Papadakos, Nader D. Nader

**Affiliations:** ^1^VA Western New York Healthcare System, Division of Critical Care Medicine, Department of Anesthesiology and Medicine, Buffalo School of Medicine and Biomedical Sciences, The State University of New York, Buffalo, NY 14215, USA; ^2^Strong Memorial Hospital, University of Rochester, Rochester, NY 14642, USA; ^3^VA Western New York Healthcare System, Division of Cardiothoracic Anesthesia and Pain Medicine, Department of Anesthesiology, Buffalo School of Medicine and Biomedical Sciences, The State University of New York, Buffalo, NY 14215, USA

## Abstract

Failure to transition patient from controlled mechanical ventilation to spontaneous breathing trials (SBTs) in a timely fashion is associated with significant morbidity and mortality in the intensive care unit. In addition, weaning failures are common in patients with limited cardiac reserves. Recent advances in cardiac echocardiography and laboratory measurement of serum biomarkers to assess hemodynamic response to SBT may provide additional information to guide clinicians to predict weaning outcome.

## 1. Introduction

Weaning critically ill patients from mechanical ventilation (MV) is a gradual and challenging process. Discontinuation of MV should be considered when patient is able to follow commands and maintain appropriate minute ventilation. In addition, protective airway reflexes should be intacts and patient clinical status must have improved. Clinical bedside assessment tools are crucial during the weaning trial (WT) so that ventilator requirements are met as the disease course is corrected. In April 2005, an international consensus conference sponsored by five major scientific societies was held in Budapest, Hungary to provide recommendations regarding the management of weaning process. The main recommendations were as follows: weaning should be considered as early as possible, patients should be divided to three categories (simple, difficult, prolonged weaning), a spontaneous breathing trial (SBT) is the major diagnostic test to determine whether patients can be successfully extubated, the initial trial should last 30 minutes and consist of either tracheal tube (T-Piece) breathing or low levels of pressure support, pressure support or assist-control ventilation modes should be favored in patients failing an initial trial/trials, and noninvasive ventilation techniques should be considered in selected patients to shorten the duration of intubation but should not be routinely used as a tool for extubation failure [1]. 

In general, mechanical weaning parameters are poor at predicting weaning success because they do not take into account cardiac reserves [2]. Therefore it is necessary for clinicians to understand the cardiovascular response to weaning trials and utilize the available tools to guide the wean team.

## 2. Physiology of Spontaneous Breathing Trials

 MV weaning trial can be compared to a cardiac stress test where spontaneous ventilation is a form of an exercise [2], and therefore hemodynamic compromise can occur during weaning process in critically ill patients. The immediate transition from positive pressure mechanical ventilation to spontaneous ventilation may generate significant cardiopulmonary alterations based on the mode of weaning selected, particularly in individuals with preexisting cardiac dysfunction. Consideration of baseline cardiac reserve may be an important factor in the selection of an appropriate mode of spontaneous ventilation following controlled MV [3]. 

There are many studies reflecting on the concept of breathing as an exercise [4–8]. The important study by Mohsenifar et al. looked at the gastric intramural pH as a predictor of success or failure in weaning patients from mechanical ventilation. He concluded that gastrointestinal acidosis may be an early sign of weaning failure resulting from low cardiac output states [9]. The study by Jubran et al. looked at mixed venous oxygen saturation (SvO_2_) monitoring for assessing hemodynamic performance and global tissue oxygenation in determining weaning outcome. He demonstrated that ventilator-supported patients who failed a trial of spontaneous breathing developed a progressive decrease in SvO_2_ caused by the combination of a relative decrease in O_2_ transport and an increase in O_2_ extraction by the tissues [10].

The original study by Lemaire and colleagues looked at the hemodynamic effects of rapidly weaning patients from MV with severe chronic obstructive pulmonary disease (COPD) and cardiovascular disease who were recovering from acute cardiopulmonary decompensation. They showed that during spontaneous breathing trials on T-piece, majority of patients demonstrated marked increase in the pulmonary artery occlusion pressure, left ventricular end diastolic volume index, and controlled MV had to be resumed [11]. Similarly, Routsi et al. demonstrated that nitroglycerin infusion can expedite the weaning by restoring weaning-induced cardiovascular compromise in COPD patients [12]. The explanation for Lemaire finding is related to the changes in lung volumes. 

**Figure 1:**
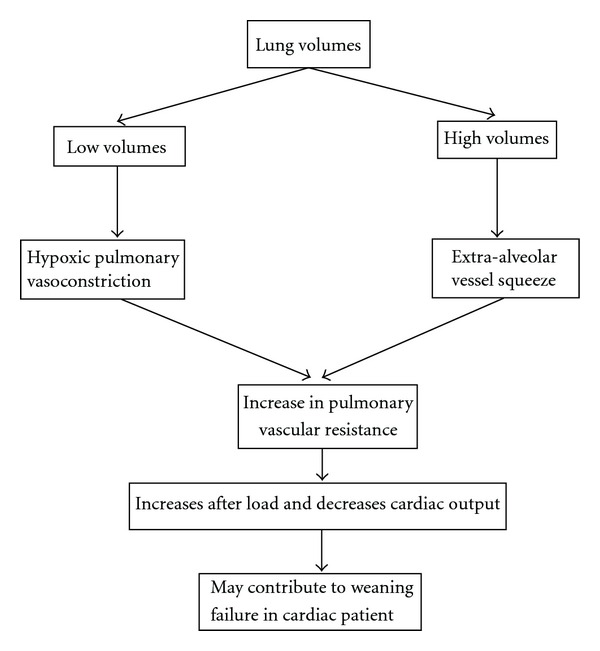


It is important to recognize the physiologic effect of lung volumes and intrathoracic pressures (ITPs) on ventilation. Low lung volumes result in alveolar collapse and hypoxia, stimulating pulmonary vasomotor tone by the process of hypoxic pulmonary vasoconstriction [13]. In contrast, at high lung volumes the increase in pulmonary vascular resistance [14] and right ventricular afterload is largely due to increase in transpulmonary pressure [15] (Figure 1). Brower et al. and other investigators concluded that during spontaneous breathing trials (SBTs), hyperventilation can lead to increase in pulmonary vascular resistance [14], and patients with lung disease are more at risk for hyperinflation and hemodynamic changes [16, 17]. Pinsky also demonstrated that changes in lung volume alter autonomic tone and pulmonary vascular resistance (PVR), and high lung volumes compress the heart in the cardiac fossa. Hyperinflation increases PVR and pulmonary artery pressure, impeding right ventricular ejection fraction [18, 19]. 

Similarly, variations in intrathoracic pressure generated by different ventilator weaning modes may significantly affect hemodynamic and cardiovascular stability [20]. Heart is an intrathoracic organ; hence any changes in intrathoracic pressures (ITPs) can lead to changes in left ventricular (LV) afterload and venous return [21, 22]. In 1984, Pinsky elegantly demonstrated in his study that spontaneous inspiratory efforts decrease intrathoracic pressure (ITP). Decreases in ITP will augment venous return and impede LV ejection and increase intrathoracic blood volume. Since diaphragmatic descent increases intra-abdominal pressure, these combined effects cause decreased right atrial pressure and increased venous pressure in the abdomen, markedly increasing the pressure gradient for systemic venous return [23]. Furthermore, the greater the decrease in ITP, the greater the increase in LV afterload for a constant arterial pressure, and right ventricle stroke output increases [24]. Pulmonary edema from negative pressure effect in patient breathing through the narrow endotracheal tube could also contribute to lower ITP. 

**Figure 2:**
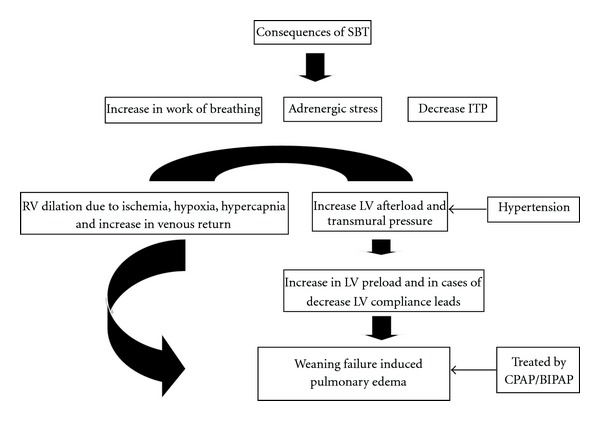
SBT: Spontaneous Breathing Trials, ITP: Intra-thoracic Pressure, CPAP: Continuous Positive Pressure Therapy, BIPAP: Bi-level Positive Pressure, LV: Left Ventricle, RV: Right Ventricle.

It is now known that loaded spontaneous inspiration leads to increase in venous return and possible decompensation to heart failure or pulmonary edema [25–27]. Other investigators have similarly shown that increases in intrathoracic pressure increase right atrial pressure and decrease transmural LV systolic pressure. This will reduce the pressure gradients for venous return and LV ejection resulting in lower thoracic blood volume. This hemodynamic alteration generates a change in autonomic tone, so that cardiac output could be maintained. Therefore, individuals with autonomic and/or cardiovascular dysfunction may not be capable of this type of response and may fail to successfully wean from mechanical ventilation [20] (Figure 2). 

## 3. Assessment of Cardiac Function during Weaning

### 3.1. B-Type Natriuretic Peptide

**Figure 3:**
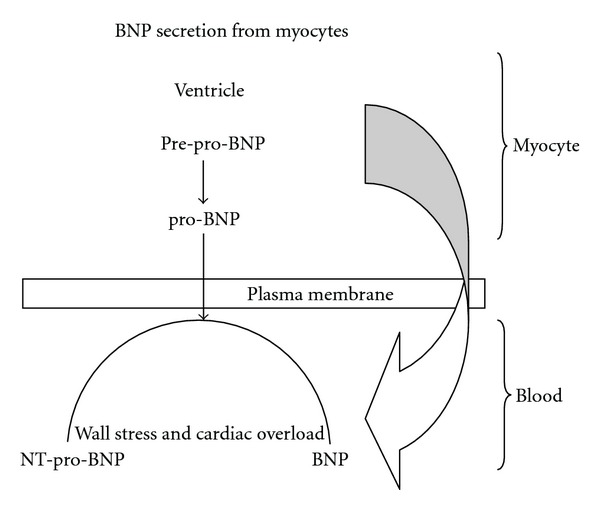
BNP: B-type natriuretic peptide, NT-pro-BNPL: N-terminal pro-B-type natriuretic peptide.

B-type natriuretic peptide levels are quantitative markers of cardiac stress and heart failure that summarize the degree of systolic and diastolic left ventricular dysfunction [28]. Initial observational pilot studies have addressed several potential indications in the intensive care unit: identification of cardiac dysfunction, diagnosis of hypoxic respiratory failure, risk stratification in severe sepsis and septic shock, evaluation of patients with shock [29], and weaning from mechanical ventilation [30]. B-type natriuretic peptide (BNP) is a cardiac neurohormone synthesized in the cardiac ventricles. It is released as a pre-pro-BNP peptide of 134 amino acids and is cleaved into pro-BNP (108 amino acids) and a signal peptide of 26 amino acids. Pro-BNP is subsequently cleaved into BNP (32 amino acids) and the inactive N-terminal pro-BNP peptide (NT-pro-BNP; 76 amino acids [Figure 3]) [31, 32]. The release of BNP into the circulation is directly proportional to the ventricular expansion and volume overload of the ventricles and therefore reflects the decompensated state of the ventricles [33, 34]. The effects of BNP—vasodilatation, natriuresis, and diuresis—lead to some improvement of the loading conditions of the failing heart. 

Multiple studies have addressed the question of whether BNP or NT-pro-BNP could be used to identify patients who fail to wean for cardiac reasons [33–40]. The study by Zapata et al. was a prospective observational study of 100 MV patients [41]. All patients underwent spontaneous breathing trials over 48 hours and were assessed by transthoracic echocardiography, pulmonary artery catheter and BNP and NT-pro-BNP. They concluded that B-type natriuretic peptides, particularly BNP, can predict weaning failure due to heart failure (HF) before an SBT. Increases in natriuretic peptides during SBT are diagnostic of HF as the cause of weaning failure. BNP performs better than NT-pro-BNP in prediction and diagnosis of HF. The cut-off values using receiver operating (ROC) curve analyses to predict HF were 263 ng/L for BNP (*P* < 0.001) and 1,343 ng/L for NT-pro-BNP (*P* = 0.08). Mekontso-Dessap et al. [37] showed BNP levels after diuretic therapy were lower in patients with weaning success (517 pg/mL versus 226 pg/mL). Grasso et al. [40] used N-terminal pro-BNP to detect acute cardiac dysfunction during weaning failure in difficult-to-wean patients with chronic obstructive pulmonary disease. He showed that plasma levels of NT-pro-BNP increased significantly at the end of the spontaneous breathing trial only in patients with acute cardiac dysfunction (median 12,733, interquartile range 16,456 pg/mL, *P* < 0.05). Chien et al. [36] used the median BNP levels after the 2 hr SBT showing BNP levels were 461 (168–1202) pg/mL, 418 (218–1085) pg/mL, and 224 (112–660) pg/mL in the SBT failure, extubation failure, and extubation success groups, respectively. Gerbaud et al. [38] prospectively evaluated 44 patients with echocardiography and NT-pro-BNP. NT-pro-BNP levels (8199 (3106–10949) versus 4200 (1855–7125) pg/mL, *P* = 0.004) increased significantly in those who failed the SBT.

### 3.2. Echocardiography

There is growing indication to advocate that transthoracic echocardiography (TTE) should be used to categorize the cardiac origin of respiratory weaning failure. The study by Gerbaud et al. looks at the weaning trials in congestive heart failure patient by analysis of the mitral Doppler inflow *E* velocity to annular tissue Doppler *E*_*a*_ wave velocity (*E*/*E*_*a*_) ratio measurement. Even though he concluded that TTE could not predict the outcome of SBT, he noticed cardiac index increased significantly at end-SBT in patients who passed [38]. In contrast, the study by Moschietto et al. in 68 patients on MV over 48 hours proved that measurement of the *E*/*E*_*a*_ ratio with TTE could predict weaning failure. Diastolic dysfunction with relaxation impairment was strongly associated with weaning failure. Additionally, the impossibility of enhancing the left ventricle relaxation rate during the SBT seemed to be the key factor of weaning failure. In contrast, the systolic dysfunction was not associated with weaning outcome [42]. Papanikolaou et al. evaluated 50 patients with Doppler echocardiography to predict outcome of weaning trials. The result indicated that LV diastolic dysfunction is significantly associated with weaning outcome in critically ill patients with preserved LV systolic function. An *E*/*E*_*a*_ ratio greater than 7.8 may identify patients at high risk of weaning failure [43]. Schifelbain et al. conducted randomized crossover clinical trial of 24 patients to analyze changes in cardiac function, using Doppler echocardiogram, in critical patients during weaning from MV. He used two different weaning methods: pressure support ventilation and T-tube. He did not find any differences between Doppler echocardiography and cardiorespiratory variables during pressure support ventilation and T-tube. However cardiac structures were smaller, isovolumetric relaxation time was larger, and oxygenation level was greater in successfully weaned patients [44]. It is probably safe to say that Doppler echocardiography has a place for assessment of weaning failure due to cardiac origin if performed routinely in the ICU. However, due to certain limitation relating to patient, it cannot be used in every patient [22, 45, 46] (Table 1). 

### 3.3. Management of Weaning Failure from Cardiac Dysfunction

Therapeutic options should take into consideration the etiology of weaning failure. Weaning failure due to excessive preload should be treated with diuretic. It is important to rule out extra cardiac causes of weaning failure in such cases. Vasodilator therapy is indicated for weaning failure due to excessive afterload or myocardial ischemia. Additionally, alteration in ITP can be prevented by the use of CPAP/BIPAP (Figure 2). Noninvasive ventilation decreases cardiac stress load and should be utilized in weaning patients with poor cardiac reserves [47, 48]. In fact, positive pressure therapy is now the standard of care for treating patients with acute pulmonary edema and decreases afterload [3, 19]. Using the same physiological concept, Marino and Langhelle et al. and others have introduced the concept of resistive loaded breathing to augment cardiac output during cardiopulmonary resuscitation [49–52]. 

## 4. Conclusion

Assessment and prediction of weaning failure from cardiac origin remain complicated. Current prediction models are difficult to implement clinically at bedside. Echocardiography remains a valuable tool to monitor respiratory weaning process and requires expertise in image interpretation. Additionally, the need for multiple assessments makes it difficult to implement echocardiography as a routine monitor in the intensive care setting. Serum BNP and NT-pro-BNP appear promising to identify patients with heart failure during weaning process. However, laboratory turnover time and the accepted cut-off values for HF pose a clinical challenge for data interpretation in the intensive care arena.

## 5. Key Messages for Practicing Intensivists

- Ischemic heart disease, valvular heart disease, systolic or diastolic dysfunction contributes to increase in cardiac load and weaning failure.
- Extra demand on cardiac working load imposed by SBT may become apparent when transferring patient from positive to spontaneous ventilation.
- Diuretic therapy may be considered for excessive preload.
- Noninvasive positive pressure ventilation is beneficial for weaning-induced pulmonary edema.
- Further cardiac evaluation is necessary if changes in natriuretic peptide levels are detected during SBT.

## Figures and Tables

**Table 1:** Prediction of respiratory weaning outcomes.

Author	Number	Study	Outcome
Zapata et al. [41]	100	Prospective	BNP or pro-BNP & ECHO predict WO
Gerbaud et al. [38]	44	Prospective	BNP or pro-BNP & ECHO did not predict WO
Papaioannou et al. [7]	42	Prospective	Cardiorespiratory dynamics predict WO
Chien et al. [36]	52	Prospective	Percent change of less than 20% in BNP predicts WO
Mekontso-Dessap et al. [37]	102	Prospective	Lower BNP levels before SBT may predicts WO
Moschietto et al. [42]	68	Prospective	ECHO predicts WO
Schifelbain et al. [44]	24	Prospective	ECHO did not predicts WO
Caille et al. [46]	117	Prospective	ECHO predicts WO
Grasso et al. [40]	19	Prospective	BNP predicts WO

ECHO: echocardiography, WO: weaning outcome, BNP: B-type natriuretic peptide.
